# Intraosseous Bone Marrow Concentrate and Long-Term Procedure-Free Survival in Bilateral Non-Traumatic Ankle Osteoarthritis: A Contralateral-Controlled Study

**DOI:** 10.3390/medicina62091693

**Published:** 2026-09-03

**Authors:** Philippe Hernigou, Christopher J. Centeno, Dustin R. Berger, Ehren Dodson, Matthew B. Murphy

**Affiliations:** 1 Faculty of Medicine, Université Paris-Est Créteil, 94010 Créteil, France; 2 Orthopaedic Department, Hôpitaux Universitaires Henri-Mondor, 94000 Créteil, France; 3 Centeno-Schultz Clinic, Broomfield, CO 80021, USA; centenooffice@centenoschultz.com; 4 Regenexx, Broomfield, CO 80021, USA; dberger@regenexx.com (D.R.B.); edodson@regenexx.com (E.D.)

**Keywords:** ankle osteoarthritis, intraosseous bone marrow concentrate, colony-forming unit-fibroblast, joint preservation, ankle arthrodesis, total ankle arthroplasty, talar osteonecrosis

## Abstract

*Background and Objectives:* Joint-preserving options for bilateral non-traumatic ankle osteoarthritis (OA) are limited. This study evaluated whether intraosseous bone marrow concentrate (IO BMC) was associated with a lower observed occurrence of ankle arthrodesis or total ankle arthroplasty and greater procedure-free survival, and whether delivered colony-forming unit-fibroblast (CFU-F) count was associated with procedure-free survival. *Materials and Methods:* This retrospective, non-randomized, contralateral-controlled study included 88 patients with bilateral non-traumatic ankle OA treated between 2000 and 2014. The more symptomatic ankle received IO BMC, whereas the contralateral ankle was managed non-operatively. The primary endpoint was subsequent ankle arthrodesis or total ankle arthroplasty. Survival analyses were truncated at 15 years. *Results:* Arthrodesis or arthroplasty occurred in 16 of 88 IO BMC-treated ankles (18.2%) and 44 of 88 control ankles (50.0%). Thirty patients underwent a procedure only on the control side compared with two only on the IO BMC-treated side (*p* < 0.001). In the adjusted Cox model, IO BMC treatment was associated with a lower hazard over 15 years (summary HR = 0.24, 95% CI 0.15 to 0.38, *p* < 0.001), representing a summary association over the analysis interval rather than a constant effect. Restricted mean procedure-free survival was 9.89 versus 8.10 years through ten years (difference 1.78 years, 95% CI 1.28 to 2.32) and 14.23 versus 10.77 years through 15 years (difference 3.46 years, 95% CI 2.58 to 4.40). In an exploratory analysis with only 16 endpoint events, higher delivered CFU-F count remained associated with greater procedure-free survival (HR = 0.70 per 10,000-cell increase, 95% CI 0.55 to 0.90, *p* = 0.004). *Conclusions:* IO BMC treatment was associated with a lower observed occurrence of arthrodesis or arthroplasty and greater procedure-free survival in bilateral non-traumatic ankle OA. Higher delivered CFU-F counts were also associated with greater procedure-free survival in an exploratory analysis. These observational findings do not establish a causal treatment effect.

## 1. Introduction

Ankle osteoarthritis (OA) is less common than hip or knee OA but can cause substantial pain, stiffness, diminished mobility, and quality-of-life impairment [1,2]. Approximately one percent of adults are affected by ankle OA [1], and patients with end-stage ankle arthrosis have been reported to have health-related quality-of-life impairment comparable to that of patients with end-stage hip arthrosis [2]. Unlike hip and knee OA, which are predominantly primary or idiopathic, ankle OA is more often secondary to prior trauma, with non-traumatic causes representing a smaller and clinically heterogeneous subset [3,4]. Non-traumatic ankle OA may be idiopathic or secondary to systemic disease or congenital deformity [1,4], may develop after talar osteonecrosis associated with corticosteroid exposure or sickle cell disease [5], or may occur in association with articular crystal deposition [6]. In bilateral disease, treatment decisions are particularly complex because loss of motion, delayed recovery, or failure on one side may directly affect the patient’s remaining functional reserve.

Osteoarthritis is a multifactorial whole-joint disorder in which mechanical loading, aging, genetic susceptibility, metabolic factors, prior injury, and systemic disease interact. Progressive cartilage matrix disruption and loss of chondrocyte homeostasis occur alongside synovial inflammation, osteophyte formation, and remodeling of the subchondral plate and trabecular bone. Cartilage and subchondral bone function as a mechanically and biologically coupled unit. Altered load transmission, microdamage, vascular changes, and bone-marrow lesions may contribute to pain and structural progression. Contemporary diagnostic approaches therefore integrate symptoms, examination, weight-bearing radiographs, and, when appropriate, advanced imaging to characterize both cartilage and osseous disease [7,8]. Initial treatment generally includes education, activity and weight modification when appropriate, analgesic or anti-inflammatory medication, bracing, physical therapy, and selected injections, with injectable orthobiologic approaches increasingly considered within the spectrum of conservative and joint-preserving ankle care [9,10]. Joint-preserving surgery, including cartilage-restoration procedures for selected osteochondral lesions, may be considered in suitable earlier-stage or mechanically correctable disease [11,12].

For advanced ankle OA that remains symptomatic despite non-operative care, the principal surgical options are ankle arthrodesis and total ankle arthroplasty [1,13]. Arthrodesis is a durable pain-relieving procedure but eliminates tibiotalar motion, may alter gait mechanics, and can increase mechanical demand on adjacent joints [13,14,15]. These tradeoffs are especially relevant when bilateral fusion is contemplated [16]. Total ankle arthroplasty preserves motion and may improve selected gait parameters compared with fusion, but concerns remain regarding implant survivorship and complications such as loosening, subsidence, infection, reoperation, and revision [13,14,17,18]. These treatment limitations are amplified when talar osteonecrosis or collapse is present, because progressive loss of talar structural integrity can narrow reconstructive options [19,20,21]. Although modern reconstructive options, including total talus replacement or combined talar and ankle reconstruction, have expanded the treatment armamentarium for severe talar disease, these approaches remain technically demanding and require further long-term validation [21,22].

Intraosseous bone marrow concentrate (IO BMC) offers a joint-preserving strategy that targets the subchondral bone compartment rather than replacing or fusing the joint. This route may be relevant in ankle OA because subchondral bone is increasingly implicated in OA pain and progression [23], providing a rationale for the targeted delivery of marrow-derived cells and signaling factors to this compartment. Bone marrow concentrate contains a heterogeneous mixture of nucleated cells, mesenchymal stromal/progenitor cells, hematopoietic-lineage cells, platelets, growth factors, cytokines, and other marrow-derived signaling factors that may collectively modulate local inflammation and tissue remodeling [24,25]. The colony-forming unit-fibroblast (CFU-F) count provides an estimate of mesenchymal stromal/progenitor-cell content, and higher transplanted CFU-F counts have been associated with improved bone-healing outcomes in prior orthopedic applications [26]. While orthobiologic approaches have been increasingly described in foot and ankle surgery, long-term clinical data evaluating IO BMC for ankle OA remain limited [9,10,25,27].

The purpose of this study was to evaluate long-term outcomes in patients with bilateral non-traumatic ankle OA in whom one ankle was treated with autologous IO BMC and the contralateral ankle was managed non-operatively as a within-patient contralateral control. Specifically, we asked whether (1) IO BMC was associated with a lower observed occurrence of a later joint-sacrificing ankle procedure, defined as ankle arthrodesis or total ankle arthroplasty, and greater procedure-free survival, (2) among ankles that ultimately required definitive surgery, the observed interval to arthrodesis or arthroplasty differed according to prior IO BMC treatment, (3) higher delivered CFU-F count was associated with greater joint-sacrificing procedure-free survival among IO BMC-treated ankles, and (4) postoperative pain and adverse-event outcomes differed according to prior IO BMC treatment when later arthrodesis or arthroplasty was required. Outcomes were further summarized by osteonecrosis status to determine whether the observed treatment association was present in both non-osteonecrosis OA and osteonecrosis-related OA.

## 2. Materials and Methods

### 2.1. Study Design

This retrospective contralateral-controlled study included patients with bilateral non-traumatic ankle OA. After symptom severity and radiographic severity were assessed in each ankle, the more symptomatic and radiographically advanced ankle was treated with autologous IO BMC, whereas the contralateral ankle was managed non-operatively and served as a within-patient contralateral control. When symptom severity and radiographic severity identified different ankles, the more symptomatic ankle was selected for treatment. The index date for both ankles was the date of unilateral IO BMC treatment, and neither ankle had undergone prior arthrodesis or total ankle arthroplasty. The study evaluated progression to later arthrodesis or arthroplasty, timing of such procedures, and postoperative outcomes. This study was conducted under a hospital registry protocol. All patients provided written informed consent for treatment and registry participation before data collection. This study is reported in accordance with the Strengthening the Reporting of Observational Studies in Epidemiology (STROBE) statement [28].

### 2.2. Patient Population

Patients were treated between January 2000 and December 2014 at a single site by the same surgical team. Clinical records were reviewed through July 2025. Eligibility criteria were bilateral non-traumatic ankle OA, Kellgren-Lawrence (KL) OA grade two or higher [29], unilateral IO BMC treatment with contralateral non-operative management, and more than ten years of clinical follow-up for both ankles. Median final clinical follow-up was 15 years (ranging from 11 to 19 years). No included patient was lost to hospital follow-up. Among 196 patients screened with a diagnosis of non-traumatic ankle OA, 128 had bilateral disease and were potentially eligible. Forty patients were excluded because of prior arthrodesis (seven cases), advanced adjacent-joint disease (five cases), infection (six cases), or inflammatory arthritis (22 cases), leaving 88 patients for analysis. No potentially eligible patients were excluded because of inadequate follow-up. No a priori sample-size calculation was performed and all eligible patients meeting the study criteria were included.

Patients were categorized as having non-osteonecrosis OA or osteonecrosis-related OA. The non-osteonecrosis OA group included 28 patients (idiopathic OA in ten, multiple epiphyseal dysplasia in six, and chondrocalcinosis/calcium pyrophosphate deposition disease in 12). The osteonecrosis-related OA group included corticosteroid-induced talar osteonecrosis in 34 patients and sickle cell disease-related talar osteonecrosis in 26 patients. Talar osteonecrosis was confirmed by radiographs and magnetic resonance imaging (MRI). All patients with osteonecrosis-related OA had bilateral talar collapse, with ankle OA developing a mean of two years after collapse (ranging from 1 to 3 years).

Among patients with corticosteroid-induced talar osteonecrosis, corticosteroid exposure was recorded as a methylprednisolone-equivalent dose. The mean cumulative dose was 7865 mg and the mean peak dose was 342 mg. Corticosteroids were discontinued when clinically possible. In the sickle cell disease-related subgroup, 19 patients were homozygous for hemoglobin S and seven had hemoglobin S/hemoglobin C disease.

Radiographic review included anteroposterior and lateral ankle weight-bearing radiographs for all ankles at presentation and most recent follow-up. KL grades were assigned by the treating site’s radiologic department. For ankles with osteonecrosis-related OA, coronal and sagittal MRI sequences were obtained at presentation. The talar dome was divided into four coronal and four sagittal sections to characterize osteonecrosis extent [19]. Lesion volume and percentage talar involvement were calculated using a previously described talar dome volume method, and involvement was graded as mild (<15%), moderate (15% to 30%), or severe (>30%) [20].

### 2.3. Treatment Protocol

Bone marrow aspirate (BMA) was obtained under general anesthesia from the anterior or posterior iliac crest. A total volume of 100 to 120 mL of BMA was aspirated using ten-mL syringes and small-volume draws of one to five mL to minimize peripheral blood dilution [30]. Syringes were rinsed with heparin solution, and the aspirate was mixed with 10% acid-citrate-dextrose solution A upon collection to prevent clot formation. Aspirations were performed from multiple sites along the selected iliac crest to maximize progenitor cell yield. The aspirate was filtered to remove cellular aggregates and fat (Hemosed NSR LP, B. Braun Melsungen AG, Melsungen, Germany) and centrifuged for five minutes at 1200× *g* (COBE 2991 cell processor, Gambro BCT, Inc., Lakewood, CO, USA) to obtain a concentrated buffy-coat fraction. Approximately 22 mL of BMC was prepared, of which 20 mL was used immediately for intraosseous injection, performed with the same trocar as for bone marrow aspiration [31].

Patients with non-osteonecrosis OA received ten mL of BMC into the talus and ten mL into the distal tibia. Patients with corticosteroid-induced osteonecrosis or sickle cell disease-related osteonecrosis in the early stages of OA (KL2), with joint-space narrowing without osteochondral lesions on the distal tibial side, received 20 mL of BMC into the talus only. At later stages, they received ten mL of BMC into the talus and ten mL of BMC into the distal tibia. Intraosseous injections were performed under fluoroscopic guidance. Approximately two mL of the BMC product was reserved for in vitro CFU-F quantification. Quadruplicate aliquots containing 2 × 10^6^ cells were inoculated into 25-cm^2^ tissue-culture flasks containing ten mL of α-minimum essential medium supplemented with 20% fetal calf serum, 1% L-glutamine, penicillin (100 U/mL), and streptomycin (100 µg/mL). Cultures were maintained at 37 °C in a humidified atmosphere containing 5% CO_2_, with complete medium replacement every three to four days. On day ten, colonies were stained with Giemsa and counted under an inverted microscope at 25× magnification. An aggregate containing more than 50 fibroblast-like cells was classified as a colony. Results were expressed as the mean number of CFU-F per 10^6^ bone marrow cells and extrapolated to estimate total CFU-F count in the injected BMC product.

Following IO BMC treatment, patients were permitted full weight bearing with two canes for eight days, although pain often limited ambulation. Most patients were able to walk without a cane or crutches after the tenth day. The contralateral ankle was managed non-operatively according to patient symptoms and treating-surgeon discretion. Conservative care included activity modification, medications, bracing, physical therapy, injections, or other non-operative measures as clinically indicated.

### 2.4. Outcomes

The primary outcome was progression to a joint-sacrificing ankle procedure, defined as ankle arthrodesis or total ankle arthroplasty. To reduce potential bias related to prior IO BMC treatment, the hospital protocol used predefined criteria for recommending definitive surgery. These protocol criteria included a Mazur score < 70 together with progression of talar collapse or progression in radiographic OA grade. The Mazur ankle score ranges from 0 to 100, with higher scores indicating better function [32]. Surgical indications were reviewed by the hospital’s orthopedic staff, which comprised 15 surgeons. No included patient underwent the endpoint procedure at another institution. At each surgical decision point, fulfillment of both the clinical and radiographic components was verified from the available clinical and radiographic record and reviewed by the orthopedic staff. All ankles that underwent arthrodesis or arthroplasty met both the clinical and radiographic protocol criteria at the time surgery was recommended. Because proceeding to surgery also depended on patient preference, medical fitness, and shared decision-making, the procedure endpoint was considered a clinical-management endpoint rather than a purely biological measure of disease progression.

When definitive surgery was indicated, the choice between arthrodesis and arthroplasty was based on surgeon judgment, patient factors, deformity, bone stock, talar viability, and adjacent-joint involvement. In osteonecrosis-related OA, procedure selection depended primarily on the extent of talar necrosis. Arthrodesis was preferred when necrosis was too extensive to provide adequate viable bone support for arthroplasty. In non-osteonecrosis OA, the choice depended primarily on deformity severity and adjacent-joint involvement.

Secondary outcomes included time from IO BMC treatment to the primary endpoint, patient-reported pain, and postoperative adverse events during available follow-up after the primary endpoint procedure, including complications, reoperation, revision, amputation, or fusion. Pain scores were recorded on a zero to ten scale, with higher scores indicating worse pain, and were collected prospectively for both ankles at baseline, six months after IO BMC treatment, at the time of any arthrodesis or arthroplasty, one year postoperatively, and at final follow-up. Pain data were complete at all applicable assessment points, with no missing observations. Six-month pain outcomes were summarized descriptively using the available aggregate mean values. Final follow-up pain was defined as the pain score recorded concurrently in both ankles at the final available follow-up visit, 11 to 19 years after IO BMC treatment. For ankles that underwent arthrodesis or arthroplasty, this value represented final postoperative pain. Patients in whom both ankles eventually underwent the primary endpoint procedure were analyzed separately as a paired bilateral subgroup.

### 2.5. Ethical and Regulatory Considerations

This retrospective study was conducted in accordance with the principles of the Declaration of Helsinki and involved only the secondary use of clinical data previously collected during routine care. As no additional intervention or research-specific procedure involving patients was performed, review by a Comité de protection des personnes (CPP) was not required under the applicable French regulatory framework. The processing of personal health data was performed in accordance with the French Data Protection Authority (Commission nationale de l’informatique et des libertés, CNIL) Reference Methodology MR-004. All patients had provided written informed consent for treatment and participation in the institutional registry before their data were collected.

### 2.6. Statistical Analysis

Continuous variables are presented as mean ± standard deviation and median [interquartile range]. Categorical variables are presented as counts and percentages. Because all included patients had more than ten years of follow-up, observed five- and ten-year joint-sacrificing procedure proportions were complete within the study cohort. Survival analyses were administratively truncated at 15 years. Ankles that did not undergo arthrodesis or arthroplasty were censored at final available follow-up or at 15 years, whichever occurred first. Clinical observations obtained after 15 years were not included in the survival analyses but were retained for final follow-up pain and postoperative outcome summaries.

Patient-level joint-sacrificing procedure patterns were summarized according to whether neither ankle, only the control ankle, only the IO BMC-treated ankle, or both ankles underwent a joint-sacrificing procedure. The discordant comparison between the control-only and IO BMC-only joint-sacrificing procedure categories was evaluated using an exact binomial test of discordant pairs. Kaplan–Meier curves were used to display joint-sacrificing procedure-free survival [33]. Cox proportional hazards models were used to estimate hazard ratios (HRs) and corresponding *p* values [34]. Models comparing IO BMC-treated ankles with contralateral control ankles used robust standard errors clustered by patient to account for the bilateral contralateral-control design [35]. A pair-stratified Cox model conditioning on matched patient pairs was performed as a sensitivity analysis. The adjusted overall Cox model included treatment group, OA subtype, KL grade, age, and body mass index (BMI). Baseline pain was not included because treatment assignment was based principally on symptom severity, making baseline pain structurally linked to treatment group. Talar involvement was assessed only in ankles with osteonecrosis-related OA and therefore was not included in the whole-cohort adjusted model. Non-osteonecrosis OA served as the reference category for OA subtype. KL grade was modeled as an ordinal numeric variable, with the HR representing a one-grade increase. Age was modeled per decade and BMI per 5 kg/m^2^. The proportional hazards assumption was assessed using scaled Schoenfeld residuals [36].

As a complementary analysis, restricted mean procedure-free survival time (RMST) was estimated as the area under the Kaplan–Meier survival curve through 10 and 15 years. RMST differences were calculated as IO BMC minus control, with positive values indicating greater procedure-free time after IO BMC. To account for the paired contralateral design, 95% confidence intervals for RMST differences were estimated using 5000 patient-level bootstrap resamples, with both ankles from each patient retained together within each resampled pair. When proportional hazards testing suggested non-proportionality, Cox HRs were interpreted as summary associations over the analysis interval rather than constant treatment effects over time and were considered alongside Kaplan–Meier and RMST estimates.

Among IO BMC-treated ankles, CFU-F count was analyzed principally as a continuous variable. An exploratory IO BMC-only Cox model evaluated CFU-F count per 10,000-cell increase after adjustment for KL grade, age, and BMI. Because only 16 endpoint events occurred in treated ankles, this model was considered potentially imprecise and unstable, and all dose–response findings were hypothesis-generating. A median split at 88 × 10^3^ CFU-F was retained only for an illustrative, unadjusted presentation. This sample-specific, data-derived value has not been externally validated and should not be interpreted as a clinical cutoff, treatment target, or minimum effective dose. CFU-F counts between ankles that did or did not progress to a joint-sacrificing procedure were compared using Mann–Whitney U tests. Fully paired pain and timing comparisons were evaluated using Wilcoxon matched-pairs signed-rank tests. Timing comparisons that included non-identical groups of ankles, including all-procedure, arthrodesis-only, and arthroplasty-only comparisons, were reported descriptively. Cox-model variables were complete, and no imputation was performed. No adjustment for multiple comparisons was performed. Except for the primary endpoint analyses, secondary, subgroup, dose–response, pain, and postoperative analyses were considered exploratory. All statistical tests were two-sided, and significance was set at *p* < 0.05. Analyses were performed using R version 4.5.1 (R Foundation for Statistical Computing, Vienna, Austria) in RStudio version 2025.09.1+401 (Posit Software, PBC, Boston, MA, USA) and GraphPad Prism version 11.0.1 (GraphPad Software, LLC, Boston, MA, USA).

### 2.7. Use of Generative Artificial Intelligence

ChatGPT (OpenAI OpCo, LLC, San Francisco, CA, USA; GPT-5.6 Thinking) was used to assist with development and review of R code, as well as presentation of statistical findings, manuscript organization, and consistency review. The statistical approach was selected by the authors, and all code, outputs, and manuscript content were reviewed and verified by the authors.

## 3. Results

### 3.1. Characteristics of Study Cohort

The study cohort included 88 patients with bilateral non-traumatic ankle OA, each contributing one IO BMC-treated ankle and one contralateral control ankle. Non-traumatic OA subtypes included non-osteonecrosis OA in 28 patients, corticosteroid-induced talar osteonecrosis in 34 patients, and sickle cell disease-related talar osteonecrosis in 26 patients. The overall mean patient age was 40.8 ± 11.1 years, and mean BMI was 25.1 ± 2.4 kg/m^2^. IO BMC-treated ankles had more advanced radiographic disease than contralateral control ankles, with KL grade four disease present in 28 of 88 IO BMC-treated ankles (31.8%) compared with 13 of 88 control ankles (14.8%) (Table 1). IO BMC-treated ankles were also more symptomatic at baseline, with mean pain scores of 9.4 compared with 6.5 in contralateral control ankles. At six months, reported mean pain scores were 3.4 in IO BMC-treated ankles and 6.5 in control ankles. Among the 60 patients with osteonecrosis-related OA, talar involvement in the IO BMC-treated ankle was moderate in 22 and severe in 38, compared with moderate involvement in 35 and severe involvement in 25 control ankles. The mean total delivered CFU-F count among IO BMC-treated ankles was 92.9 ± 33.3 × 10^3^.

### 3.2. Progression to a Joint-Sacrificing Ankle Procedure

**Figure 1:**
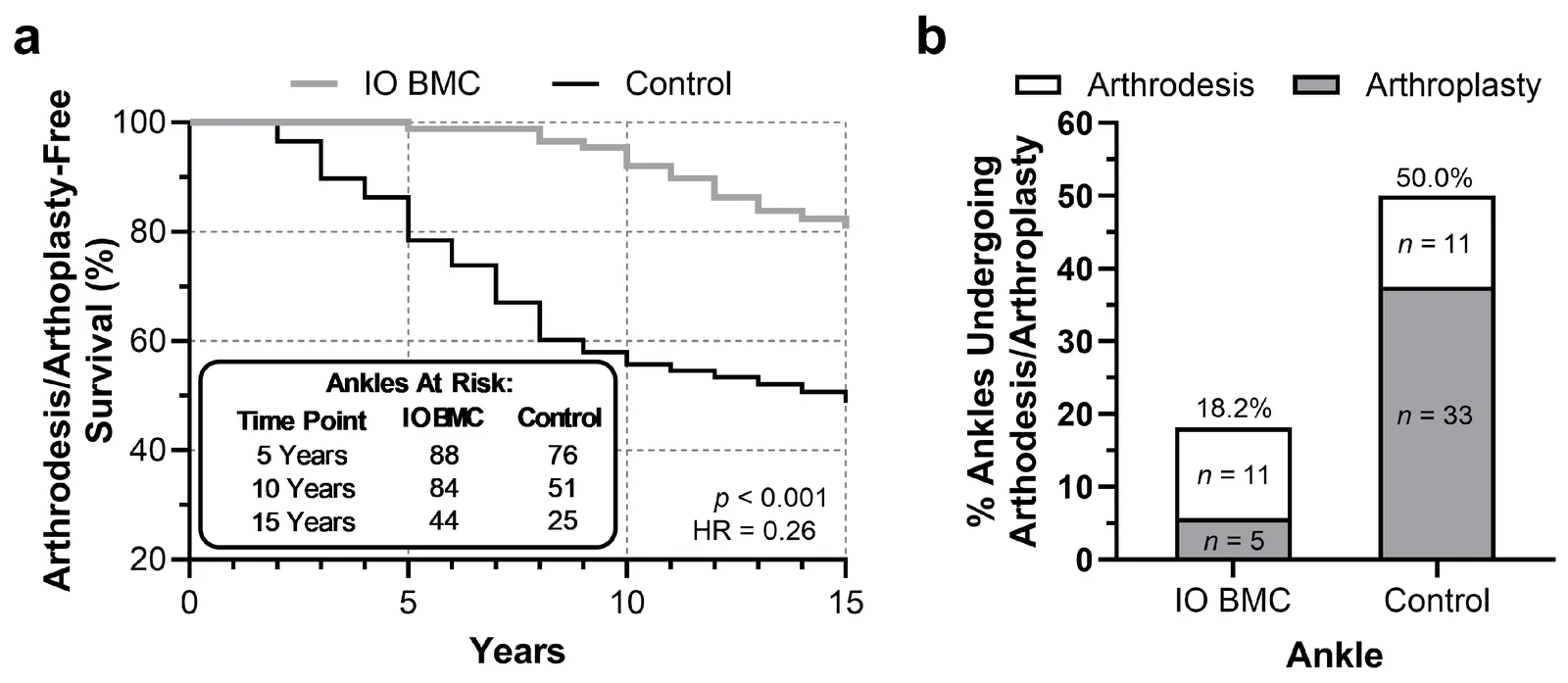
Joint-sacrificing procedure-free survival and observed procedure proportions. (**a**) Kaplan–Meier curves through 15 years. Ankles without arthrodesis or arthroplasty were censored at final follow-up or 15 years, whichever occurred first. The HR and *p* value are from an unadjusted Cox model with robust standard errors clustered by patient. Because treatment violated proportional hazards, the HR is a summary association over 15 years. (**b**) Crude observed proportions through 15 years, stratified by procedure type. The 16 IO BMC-treated and 44 control procedures included 14 patients who underwent arthrodesis or arthroplasty in both ankles.

Overall, 60 ankles underwent arthrodesis or total ankle arthroplasty during the 15-year survival-analysis period, including 16 of 88 IO BMC-treated ankles and 44 of 88 contralateral control ankles. The corresponding crude observed proportions were 18.2% and 50.0%, respectively, and Kaplan–Meier analysis demonstrated higher arthrodesis- or arthroplasty-free survival in IO BMC-treated ankles than in control ankles (Figure 1). At ten years, RMST was 9.89 years in IO BMC-treated ankles and 8.10 years in control ankles, corresponding to 1.78 additional procedure-free years associated with IO BMC (95% CI 1.28 to 2.32). At 15 years, RMST was 14.23 and 10.77 years, respectively, corresponding to a difference of 3.46 procedure-free years (95% CI 2.58 to 4.40). Ankle-level procedure outcomes at five and ten years, crude observed outcomes through available follow-up limited to 15 years, and follow-up status among ankles without an observed procedure are summarized (Table 2). The treatment association was significant in the unadjusted Cox model using robust standard errors clustered by patient (summary HR = 0.26, 95% CI 0.17 to 0.41, *p* < 0.001). At the patient level, 42 patients underwent neither procedure, 30 underwent a procedure only in the control ankle, two underwent a procedure only in the IO BMC-treated ankle, and 14 underwent procedures in both ankles (Table 3). The discordant comparison of 30 control-only and two IO BMC-treated-only procedures was significant by exact binomial test (*p* < 0.001).

### 3.3. Association Between CFU-F Count and Progression to a Joint-Sacrificing Ankle Procedure

**Figure 2:**
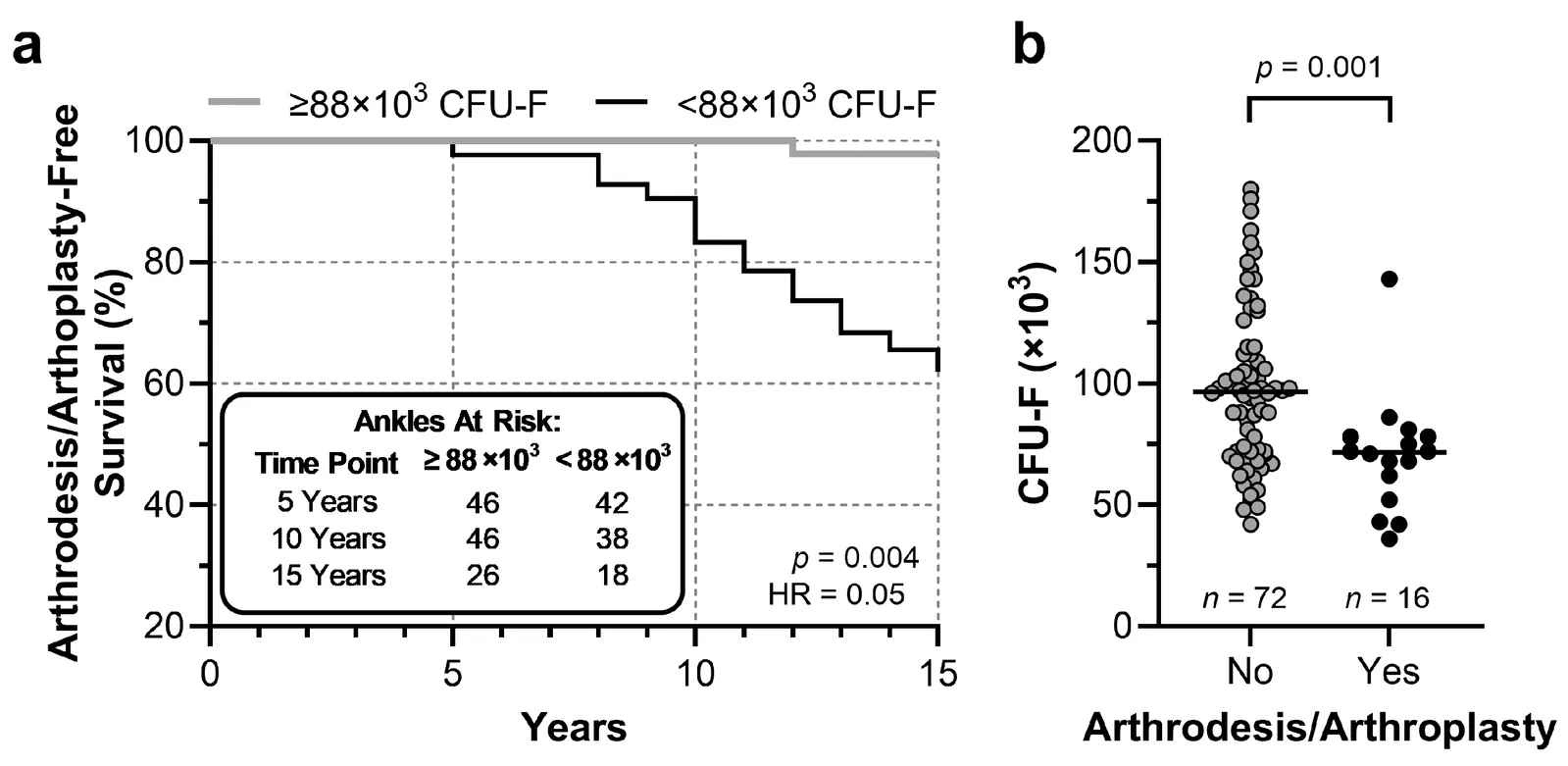
Exploratory CFU-F analyses among IO BMC-treated ankles. (**a**) Kaplan–Meier curves through 15 years, stratified at the cohort median of 88 × 10^3^ CFU-F. This sample-specific value is illustrative and is not a validated clinical threshold. The HR, 95% CI, and *p* value are from unadjusted Cox regression. (**b**) Delivered CFU-F counts in ankles with and without subsequent arthrodesis or arthroplasty through 15 years. Individual observations are shown and horizontal lines indicate medians. The unpaired comparison was analyzed using the Mann–Whitney U test. All findings are exploratory and hypothesis-generating.

In an exploratory analysis, higher total delivered CFU-F counts were associated with procedure-free survival among IO BMC-treated ankles (Figure 2). When stratified using the cohort median CFU-F threshold of 88 × 10^3^ as an illustrative unadjusted display, only one of 46 ankles (2.2%) with a CFU-F count ≥ 88 × 10^3^ progressed to a joint-sacrificing procedure, compared with 15 of 42 ankles (35.7%) with CFU-F counts < 88 × 10^3^ (HR for ≥88 × 10^3^ versus <88 × 10^3^ CFU-F = 0.05, 95% CI 0.01 to 0.38, *p* = 0.004). This sample-derived threshold is not a validated clinical cutoff or treatment target. CFU-F counts were also lower among IO BMC-treated ankles that underwent arthrodesis or arthroplasty than among those that remained free from a joint-sacrificing procedure, with counts of 70.4 ± 24.4 × 10^3^ versus 97.9 ± 33.0 × 10^3^ CFU-F, respectively (*p* = 0.001). With only 16 endpoint events, all CFU-F findings are hypothesis-generating.

### 3.4. Timing of Ankle Arthrodesis or Arthroplasty

**Figure 3:**
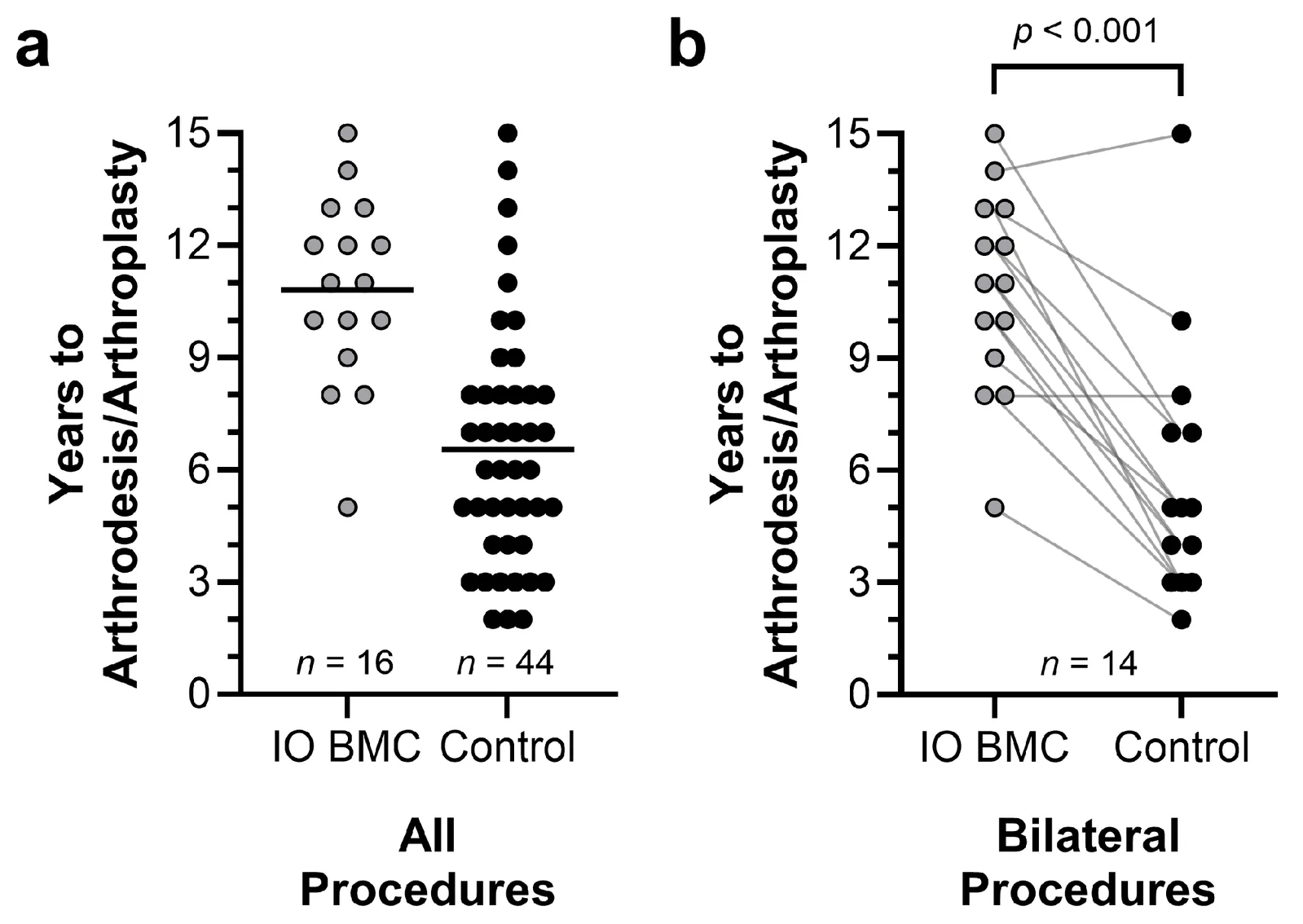
Time to ankle arthrodesis or arthroplasty from IO BMC treatment. (**a**) All ankles undergoing a joint-sacrificing procedure. The comparison is descriptive because groups are non-identical. Horizontal lines indicate means. (**b**) Paired bilateral subgroup with lines connecting ankles within patients. The paired comparison was analyzed using the Wilcoxon matched-pairs signed-rank test. Because this subgroup was restricted to patients in whom both ankles ultimately underwent a procedure, the timing comparison is descriptive.

For ankles that ultimately underwent a joint-sacrificing procedure, the observed interval from IO BMC treatment to surgery was longer in IO BMC-treated ankles than in control ankles (Figure 3 and Table 4), with mean times of 10.8 ± 2.5 and 6.5 ± 3.2 years, respectively. This event-only comparison conditions on a future event, involves non-identical groups, and is descriptive. It does not estimate a treatment-induced delay in the full cohort. When procedure type was considered separately, the same directional pattern was observed descriptively for both arthrodesis and arthroplasty. Arthrodesis occurred at a mean of 10.7 ± 2.0 years in IO BMC-treated ankles versus 5.7 ± 2.9 years in control ankles. Arthroplasty occurred at a mean of 11.0 ± 3.8 years versus 6.8 ± 3.3 years, respectively.

The 14 patients in whom both ankles eventually underwent arthrodesis or arthroplasty provided a within-patient timing comparison. Within this paired bilateral subgroup, the IO BMC-treated ankle underwent definitive surgery later in 12 patients, at the same time in one patient, and earlier in one patient. Mean timing was 10.8 ± 2.7 years for the IO BMC-treated ankle and 5.8 ± 3.5 years for the control ankle. The mean within-patient difference in observed procedure timing was 5.0 years, with the IO BMC-treated ankle undergoing the procedure later (*p* < 0.001). This paired event-conditioned comparison was considered descriptive.

### 3.5. Adverse Events Following Joint-Sacrificing Ankle Procedures

Postoperative adverse events during available follow-up were recorded in 17 of 44 control ankles and none of the 16 IO BMC-treated ankles (Table 5). Postoperative adverse-event endpoints were summarized descriptively because postoperative follow-up duration and the distribution of arthrodesis and arthroplasty differed between groups. Among these 17 control ankles, three were probable nonunions after arthrodesis without revision, four were implant-loosening events after arthroplasty without revision, and ten required a subsequent operative intervention, including revision, arthroplasty, arthrodesis, or amputation.

### 3.6. Pain Outcomes After a Joint-Sacrificing Ankle Procedure and at Final Follow-Up

Pain outcomes among ankles that underwent arthrodesis or arthroplasty are summarized descriptively (Table 6). In this subset, all ankles had a preoperative pain score of ten. One-year postoperative pain averaged 4.5 ± 1.0 in control ankles compared with 3.4 ± 0.6 in IO BMC-treated ankles. Final postoperative pain averaged 5.3 ± 1.4 in control ankles compared with 3.4 ± 0.6 in IO BMC-treated ankles. Among ankles that underwent arthrodesis or arthroplasty, the mean postoperative follow-up was 8.4 ± 3.0 years in control ankles and 3.3 ± 1.7 years in IO BMC-treated ankles. Because the groups were non-identical and underwent surgery at different times, between-group values were considered descriptive.

**Figure 4:**
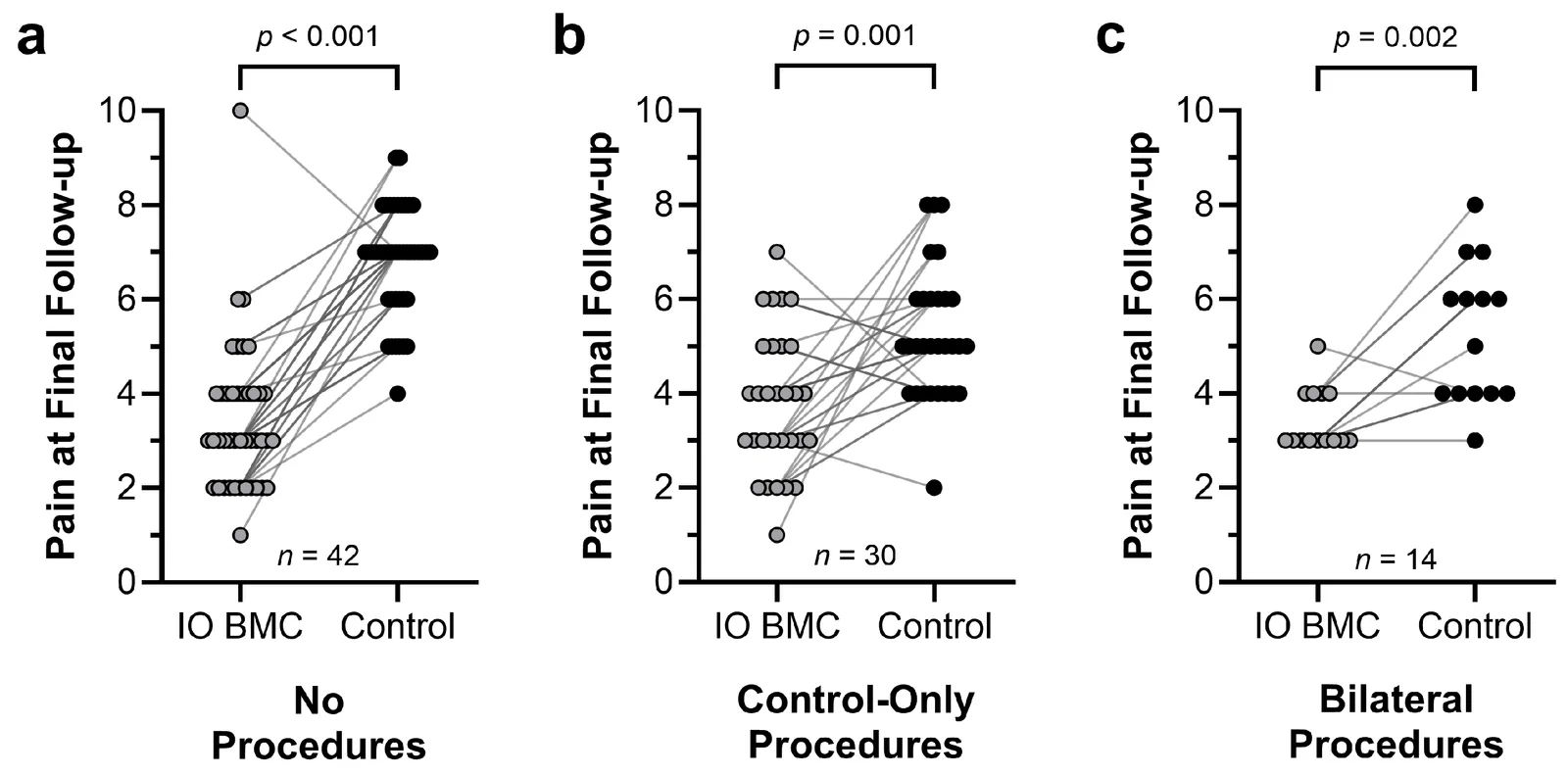
Pain in paired subgroups at the concurrent final visit, 11 to 19 years after IO BMC. Patients in whom (**a**) neither ankle, (**b**) only the control ankle, and (**c**) both ankles underwent a joint-sacrificing procedure. Lines connect ankles within patients. The IO BMC-only subgroup (*n* = 2) was not considered separately. Comparisons were analyzed using Wilcoxon matched-pairs signed-rank tests.

Final follow-up pain was lower in IO BMC-treated ankles across the three analyzed paired clinical subgroups (Figure 4). Among patients in whom neither ankle reached the primary endpoint, mean final pain was 3.4 ± 1.6 in IO BMC-treated ankles compared with 6.8 ± 1.2 in contralateral control ankles (*n* = 42 pairs, *p* < 0.001). Among patients who underwent arthrodesis or arthroplasty only on the control side, mean pain at the final paired follow-up visit was 3.8 ± 1.5 in the IO BMC-treated ankle compared with 5.3 ± 1.4 in the control ankle after surgery (*n* = 30 pairs, *p* = 0.001). Among patients in whom both ankles underwent arthrodesis or arthroplasty, mean final postoperative pain recorded concurrently was 3.4 ± 0.6 in the IO BMC-treated ankle compared with 5.3 ± 1.5 in the control ankle (*n* = 14 pairs, *p* = 0.002). Pain was lower in the IO BMC-treated ankle in 11 patients, equal in two, and higher in one.

### 3.7. Outcomes by Osteonecrosis Status

As an exploratory subgroup analysis, outcomes were also summarized by osteonecrosis status because OA subtype was clinically relevant (Table 7). Joint-sacrificing procedures occurred less often after IO BMC than in controls in both non-osteonecrosis OA and osteonecrosis-related OA at ten years and through available follow-up limited to 15 years. At ten years, observed proportions were one of 28 (3.6%) versus eight of 28 (28.6%) in non-osteonecrosis OA and six of 60 (10.0%) versus 31 of 60 (51.7%) in osteonecrosis-related OA. Through available follow-up limited to 15 years, the corresponding crude observed proportions were four of 28 (14.3%) versus eight of 28 (28.6%) and 12 of 60 (20.0%) versus 36 of 60 (60.0%).

### 3.8. Adjusted Predictors of a Joint-Sacrificing Ankle Procedure

**Figure 5:**
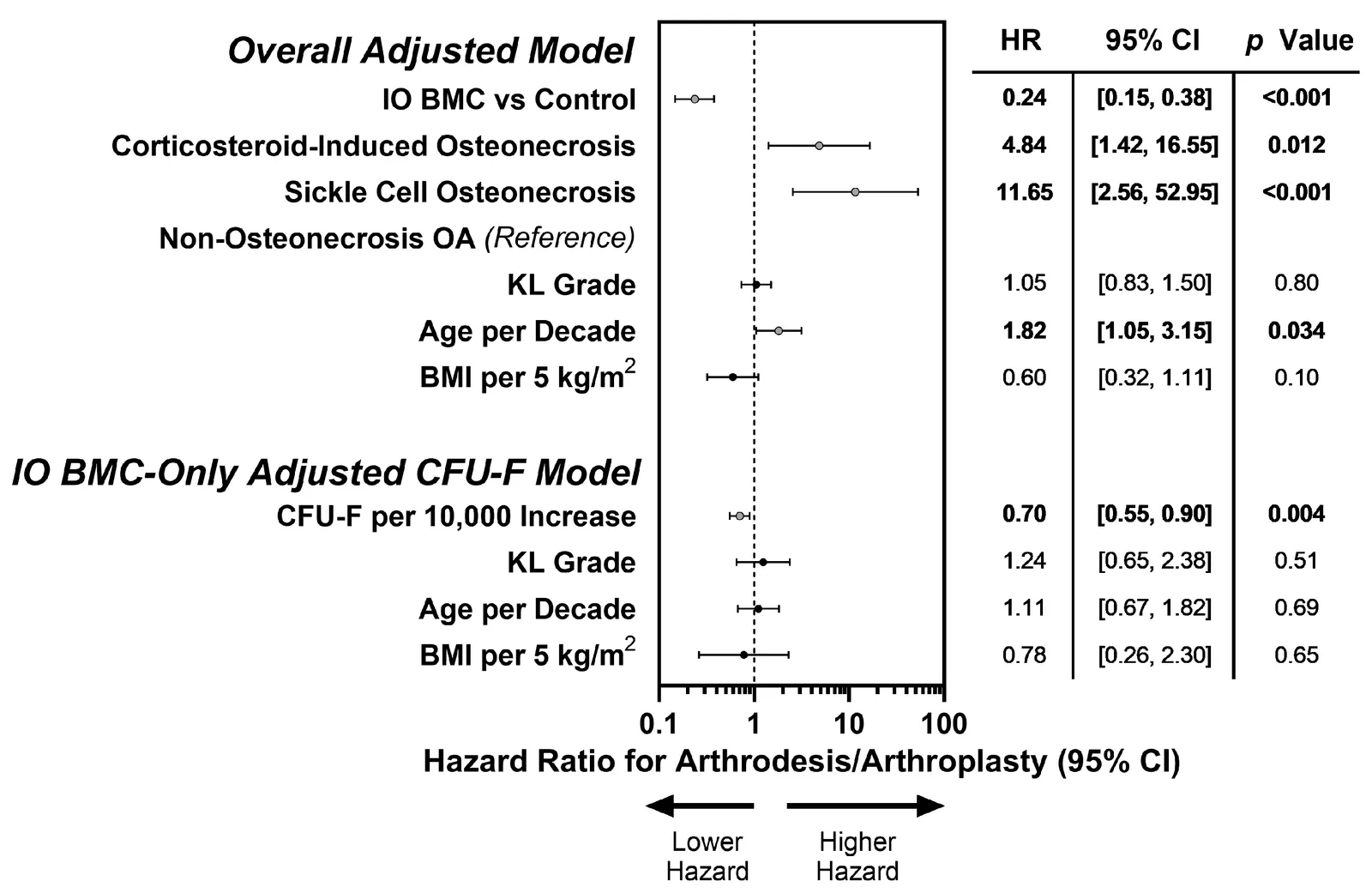
Adjusted predictors of arthrodesis or arthroplasty through 15 years. The overall model used robust standard errors clustered by patient and included treatment, OA subtype, KL grade, age, and BMI. The IO BMC-only model evaluated CFU-F after adjustment for KL grade, age, and BMI. KL grade was modeled per one-grade increase, age per decade, BMI per 5 kg/m^2^, and CFU-F per 10,000-cell increase. Error bars indicate 95% CIs. HRs for treatment, OA subtype, and KL grade in the overall model and KL grade in the IO BMC-only model are summary associations over 15 years.

Adjusted Cox regression models evaluated predictors of progression to the primary endpoint, including an overall treatment-and-covariate model and an IO BMC-only model evaluating CFU-F count (Figure 5). In the adjusted overall model using robust standard errors clustered by patient, IO BMC treatment remained associated with a lower hazard of progression to arthrodesis or arthroplasty compared with contralateral control treatment (summary HR = 0.24, 95% CI 0.15 to 0.38, *p* < 0.001). Older age was also associated with an increased hazard of progression (HR = 1.82 per decade, 95% CI 1.05 to 3.15, *p* = 0.034). Compared with non-osteonecrosis OA, corticosteroid-induced osteonecrosis (*p* = 0.012) and sickle cell disease-related osteonecrosis (*p* < 0.001) were associated with an increased hazard of progression. In a pair-stratified Cox sensitivity analysis conditioning on patient pair, IO BMC treatment remained associated with a lower hazard of progression (summary HR = 0.09, 95% CI 0.03 to 0.26, *p* < 0.001). In the exploratory IO BMC-only model, increasing CFU-F count remained associated with a reduced hazard of progression after adjustment for KL grade, age, and BMI (HR = 0.70, 95% CI 0.55 to 0.90, *p* = 0.004), although this model was interpreted cautiously because of the limited number of endpoint events among IO BMC-treated ankles (*n* = 16).

Scaled Schoenfeld residual testing indicated non-proportional hazards for treatment in the unadjusted patient-clustered model (*p* < 0.001) and the pair-stratified model (*p* = 0.006). In the adjusted overall model, non-proportionality was detected for treatment group, OA subtype, and KL grade, with a significant global test (*p* < 0.001). The corresponding HRs were therefore interpreted as summary associations over 15 years rather than constant effects and should be considered together with the Kaplan–Meier and RMST results. In the IO BMC-only model, continuous CFU-F count did not violate the proportional-hazards assumption (*p* = 0.26). Although non-proportionality was detected for KL grade (*p* = 0.015), the global test was nonsignificant (*p* = 0.13).

## 4. Discussion

The principal finding of this study was that IO BMC treatment was associated with a lower observed occurrence of arthrodesis or arthroplasty and greater procedure-free survival in patients with bilateral non-traumatic ankle OA. Because treatment violated the proportional-hazards assumption, the Cox HRs represent summary associations rather than constant effects over time. The complementary RMST analysis, which does not require proportional hazards and incorporates the complete survival experience of the cohort, showed 1.78 additional procedure-free years through ten years and 3.46 additional procedure-free years through 15 years in IO BMC-treated ankles compared with contralateral controls. The longer interval observed among ankles that ultimately underwent surgery was directionally consistent but remains a secondary descriptive comparison and should not be interpreted as the amount by which treatment delayed surgery in the overall cohort. The IO BMC-treated ankle also had greater baseline symptom severity and more advanced radiographic disease. Higher delivered CFU-F count was also associated with a lower hazard of subsequent arthrodesis or arthroplasty. Together, these findings support further evaluation of IO BMC as a potential joint-preserving strategy associated with greater procedure-free survival and a lower observed occurrence of arthrodesis or arthroplasty.

An exploratory secondary finding was the association between higher delivered CFU-F count and greater joint-sacrificing procedure-free survival. CFU-F assays estimate clonogenic marrow-derived stromal progenitor-cell content, and higher transplanted CFU-F numbers have previously been associated with improved bone-healing outcomes [26]. In the present cohort, this association remained after adjustment for KL grade, age, and BMI. The continuous CFU-F analysis provides the stronger statistical assessment because it preserves the full range of values and avoids information loss associated with dichotomization [37]. The median-based analysis at 88 × 10^3^ CFU-F illustrated the association but was derived from this cohort and should not be interpreted as an established clinical cutoff or treatment target. Moreover, CFU-F count may also reflect overall marrow quality or patient biology.

Greater procedure-free time may be clinically relevant in bilateral ankle OA. Arthrodesis can provide durable pain relief but eliminates tibiotalar motion, alters gait mechanics, and may increase mechanical demand on adjacent joints, whereas total ankle arthroplasty preserves motion but introduces risks related to implant fixation, wear, loosening, infection, reoperation, and revision [1,13,14,15,16,17,18]. These tradeoffs are amplified when both ankles are affected because neither side can be considered an unaffected compensatory limb. Postoperative pain and adverse events were summarized descriptively, as unequal surveillance duration, procedure mix, and small group sizes preclude comparative efficacy or safety conclusions.

The subchondral bone compartment provides a biologically plausible rationale for IO BMC because it contributes to OA pain and progression and is directly affected by impaired bone viability and structural support in talar osteonecrosis [19,20,21,23]. Intraosseous treatment delivers marrow-derived cells and signaling factors directly to the talar and distal tibial subchondral compartments. Prior studies have reported favorable structural or clinical outcomes after percutaneous marrow-derived cell treatment for early talar or ankle osteonecrosis, although those populations differed from patients with established non-traumatic ankle OA [20,38]. Long-term improvement has also been reported after debridement and scaffold-based BMC implantation for focal talar osteochondral lesions in osteoarthritic ankles, although the concomitant surgery and scaffold limit direct comparison with the percutaneous IO BMC approach used in the present study [39]. More broadly, ankle BMC studies have primarily addressed focal osteochondral lesions treated with concomitant cartilage procedures and remain heterogeneous in technique and study quality [10,39,40]. The present findings provide long-term clinical evidence regarding percutaneous IO BMC in ankle OA, although the study was not designed to determine whether treatment resulted in talar regeneration or structural disease modification.

The contralateral-controlled design reduced confounding from shared patient-level characteristics, including age, systemic disease, corticosteroid exposure, activity level, and comorbidity. The paired subgroup in which both ankles underwent a joint-sacrificing procedure provided an additional within-patient descriptive comparison of procedure timing and postoperative pain. Nevertheless, treatment assignment was not randomized and was based on patient-rated symptom severity, with the more symptomatic ankle receiving IO BMC. Treated ankles also had more advanced radiographic disease on average, creating potential confounding by indication. Although the treated ankle was more symptomatic and radiographically advanced at baseline, the non-randomized treatment assignment means that the direction and magnitude of residual confounding cannot be determined. Adjustment for OA subtype, KL grade, age, and BMI and the pair-stratified sensitivity analysis were consistent with the observed treatment association but cannot eliminate residual differences in deformity, lesion location, mechanical loading, or bone viability. In the adjusted overall model, proportional-hazards testing indicated that the relative effects of treatment group, OA subtype, and KL grade varied over follow-up. The corresponding HRs should therefore be interpreted as summary associations over the 15-year follow-up and considered alongside the Kaplan–Meier curves, RMST estimates, cumulative procedure proportions, and discordant-pair findings.

The cohort included idiopathic OA, multiple epiphyseal dysplasia, crystal-associated arthropathy, corticosteroid-induced talar osteonecrosis, and sickle cell disease-related talar osteonecrosis. These conditions differ in pathophysiology, natural history, bone viability, and likelihood of progression [3,4,5,6,19,20,21]. Osteonecrosis-related subtypes were associated with a greater hazard of arthrodesis or arthroplasty than non-osteonecrosis OA, although the direction of the IO BMC association was similar in both broad disease categories. With only 16 endpoints on the IO BMC-treated side, reliable CFU-F-by-etiology interaction analysis was not feasible, and subtype-specific dose–response conclusions were avoided. These observations support prospective evaluation of IO BMC as a potential joint-preserving strategy, particularly in bilateral disease and talar osteonecrosis, but do not establish that it delays or prevents surgery.

Several limitations should be acknowledged. This was a retrospective, non-randomized, single-center study conducted by one surgical team. The more symptomatic and generally more radiographically advanced ankle received IO BMC, creating substantial confounding by indication. The within-patient design and adjusted analyses cannot eliminate residual ankle-level confounding. Baseline pain was structurally linked to treatment assignment and therefore was not included as a covariate, while standardized measures of deformity, lesion location, mechanical loading, and bone viability were not consistently available for adjustment. Non-operative management of the control ankle was individualized. The endpoint reflected not only clinical and radiographic indications, but also patient preference, medical fitness, surgeon judgment, and changes in practice over time. The proportional-hazards assumption was violated for treatment in the Cox analyses. Accordingly, Cox HRs were interpreted only as summary associations over follow-up, while RMST was included to provide an absolute measure of procedure-free time that does not require proportional hazards. The timing comparison was restricted to ankles that underwent surgery and therefore conditioned on a future event. It compared non-identical subsets and was descriptive rather than an estimate of treatment-induced delay. Radiographic observer reliability was not formally assessed. The heterogeneous cohort, only 16 endpoints among treated ankles, data-derived CFU-F threshold, lack of multiplicity adjustment, and small postoperative samples make secondary, subgroup, dose–response, pain, and complication analyses exploratory. Control ankles had longer postoperative surveillance and the procedure mix differed, precluding comparative safety or efficacy conclusions. Arthrodesis and arthroplasty were combined despite different indications and failure mechanisms, and some ankles without an endpoint were censored before 15 years. Serial quantitative imaging and mechanistic biomarkers were unavailable. Strengths included long-term follow-up, the paired contralateral design, predefined criteria for definitive surgery, inclusion of challenging osteonecrosis-related disease, and characterization of the delivered biologic product [41].

## 5. Conclusions

In this retrospective contralateral-controlled cohort of patients with bilateral non-traumatic ankle OA, IO BMC treatment was associated with a lower observed occurrence of arthrodesis or arthroplasty and greater procedure-free time than that observed in contralateral non-operatively managed ankles. Treatment assignment was non-random, with the more symptomatic ankle receiving IO BMC. Moreover, treated ankles were also generally more radiographically advanced, and residual ankle-level confounding cannot be excluded. Accordingly, the findings do not establish a causal treatment effect. The exploratory association between higher delivered CFU-F count and greater procedure-free survival requires independent prospective validation. These observations support further prospective evaluation of IO BMC in bilateral non-traumatic ankle OA.

## Figures and Tables

**Table 1:** Patient characteristics and baseline ankle severity.

Characteristic	Overall	IO BMC Ankle	Control Ankle
Patients *n* (%)	Female: 40 (45.5%) Male: 48 (54.5%)	-	-
Ankles	176	88	88
Age years	40.8 ± 11.1 39.0 [31.0, 50.8]	-	-
BMI kg/m^2^	25.1 ± 2.4 25.0 [23.0, 27.0]	-	-
OA Subtype *n* (%)	Non-Osteonecrosis: 28 (31.8%) Corticosteroid Osteonecrosis: 34 (38.6%) Sickle Cell Osteonecrosis: 26 (29.5%)	-	-
KL Grade *n* (%)	-	KL2: 24 (27.3%) KL3: 36 (40.9%) KL4: 28 (31.8%)	KL2: 46 (52.3%) KL3: 29 (33.0%) KL4: 13 (14.8%)
Baseline Pain	-	9.4 ± 0.7 9.5 [9.0, 10.0]	6.5 ± 1.2 7.0 [6.0, 7.0]
Talar Involvement *n* (%)	60	Moderate: 22 (36.7%) Severe: 38 (63.3%)	Moderate: 35 (58.3%) Severe: 25 (41.7%)
Delivered CFU-F ×10^3^	-	92.9 ± 33.3 88.0 [68.0, 108.3]	-

Values are mean ± standard deviation, median [interquartile range], or *n* (%). Age, BMI, and OA subtype are patient-level. Other data are ankle-level. Talar involvement percentages are based on 60 osteonecrosis-related ankles in each treatment group.

**Table 2:** Joint-sacrificing procedure outcomes through 15 years and pain at final follow-up.

Treatment Group	Ankles *n*	Observed Procedure			No Procedure ≥15-Yr Follow-up *n* (%)	No Procedure Censored <15-Yr *n* (%)	Final Pain Score
		5-Yr *n* (%)	10-Yr *n* (%)	≤15-Yr *n* (%)			
IO BMC	88	1 (1.1%)	7 (8.0%)	16 (18.2%)	43 (48.9%)	29 (33.0%)	3.6 ± 1.4 3.0 [3.0, 4.0]
Control	88	19 (21.6%)	39 (44.3%)	44 (50.0%)	24 (27.3%)	20 (22.7%)	6.0 ± 1.5 6.0 [5.0, 7.0]

Values are *n* (%), mean ± standard deviation, or median [interquartile range]. Five- and ten-year values are complete observed proportions. Values through 15 years are crude observed proportions, not Kaplan–Meier estimates. Ankles without surgery and <15 years of follow-up were censored at the final observed visit. Final pain includes postoperative scores after arthrodesis or arthroplasty.

**Table 3:** Patient-level pattern of subsequent arthrodesis or total ankle arthroplasty.

Procedure Pattern	Patients *n* (%)
Neither ankle	42 (47.7%)
Control ankle only	30 (34.1%)
IO BMC-treated ankle only	2 (2.3%)
Both ankles	14 (15.9%)

The control-only and IO BMC-treated-only categories constituted the discordant pairs and were compared using an exact binomial test (*p* < 0.001).

**Table 4:** Time to arthrodesis or arthroplasty among ankles undergoing a joint-sacrificing procedure.

Timing Comparison	Control Ankle Years	IO BMC Ankle Years	Mean Difference Years	Statistical Comparison
All Joint-Sacrificing Procedures	*n* = 44 6.5 ± 3.2 6.0 [4.0, 8.0]	*n* = 16 10.8 ± 2.5 11.0 [9.3, 12.8]	+4.3	Descriptive
Arthrodesis Only	*n* = 11 5.7 ± 2.9 5.0 [4.0, 7.0]	*n* = 11 10.7 ± 2.0 11.0 [9.0, 12.0]	+5.0	Descriptive
Arthroplasty Only	*n* = 33 6.8 ± 3.3 7.0 [4.5, 8.5]	*n* = 5 11.0 ± 3.8 12.0 [7.5, 14.0]	+4.2	Descriptive
Paired Bilateral Joint-Sacrificing Procedures	*n* = 14 5.8 ± 3.5 5.0 [3.0, 7.3]	*n* = 14 10.8 ± 2.7 11.0 [8.8, 13.0]	+5.0	Wilcoxon matched-pairs signed-rank test, *p* < 0.001

Values are years from IO BMC treatment and are presented as mean ± standard deviation and median [interquartile range]. For the first three comparisons, values represent differences between group means. The bilateral-procedure value represents the mean within-patient difference.

**Table 5:** Postoperative adverse events after a joint-sacrificing ankle procedure.

Adverse Event Category	Control Ankle (*n* = 44)	IO BMC Ankle (*n* = 16)
Any postoperative adverse event	17 of 44 (38.6%)	0 of 16 (0%)
Probable nonunion after arthrodesis without revision	3 of 11 (27.3%)	0 of 11 (0%)
Implant loosening after arthroplasty without revision	4 of 33 (12.1%)	0 of 5 (0%)
Subsequent revision, arthroplasty, arthrodesis, or amputation	10 of 44 (22.7%)	0 of 16 (0%)

Nonunion and implant-loosening events are procedure-specific subcategories.

**Table 6:** Pain and follow-up among ankles undergoing a joint-sacrificing procedure.

Outcome or Follow-Up Measure	Control Ankle (*n* = 44)	IO BMC Ankle (*n* = 16)
Preoperative pain	10.0 10.0 [10.0, 10.0]	10.0 10.0 [10.0, 10.0]
One-year postoperative pain	4.5 ± 1.0 4.0 [4.0, 5.0]	3.4 ± 0.6 3.0 [3.0, 4.0]
Final postoperative pain	5.3 ± 1.4 5.0 [4.0, 6.0]	3.4 ± 0.6 3.0 [3.0, 4.0]
Time from IO BMC treatment to final follow-up	15.0 ± 1.8 years 15.0 [14.0, 16.0]	14.1 ± 1.5 years 14.0 [13.0, 15.0]
Time from joint-sacrificing procedure to final follow-up	8.4 ± 3.0 years 9.0 [7.0, 11.0]	3.3 ± 1.7 years 3.0 [2.0, 4.0]

Values are mean ± standard deviation and median [interquartile range].

**Table 7:** Joint-sacrificing procedures and final pain by osteonecrosis status.

Osteonecrosis Status	Treatment Group	Ankles *n*	10-Yr Procedure *n* (%)	Observed ≤15-Yr Procedure *n* (%)	Final Pain Score
Non-Osteonecrosis OA	IO BMC	28	1 of 28 (3.6%)	4 of 28 (14.3%)	3.2 ± 1.8 3.0 [2.0, 4.0]
Non-Osteonecrosis OA	Control	28	8 of 28 (28.6%)	8 of 28 (28.6%)	6.4 ± 1.4 7.0 [5.3, 7.0]
Osteonecrosis- Related OA	IO BMC	60	6 of 60 (10.0%)	12 of 60 (20.0%)	3.7 ± 1.2 4.0 [3.0, 4.0]
Osteonecrosis- Related OA	Control	60	31 of 60 (51.7%)	36 of 60 (60.0%)	5.9 ± 1.5 6.0 [5.0, 7.0]

Values are *n* (%), mean ± standard deviation, and median [interquartile range]. Osteonecrosis-related OA includes corticosteroid-induced and sickle cell disease-related osteonecrosis. Ten-year values are complete observed proportions. Values through 15 years are crude observed proportions and are not Kaplan–Meier estimates. Final pain is the final available score, including postoperative pain after arthrodesis or arthroplasty.

## Data Availability

The data supporting the findings of this study are not publicly available because they contain potentially identifiable clinical information but are available from the corresponding author upon reasonable request, subject to applicable ethical and data-protection requirements.

## Abbreviations

The following abbreviations are used in this manuscript:

BMI	body mass index
BMA	bone marrow aspirate
BMC	bone marrow concentrate
CFU-F	colony-forming unit-fibroblast
CI	confidence interval
HR	hazard ratio
IO BMC	intraosseous bone marrow concentrate
KL	Kellgren-Lawrence
MRI	magnetic resonance imaging
OA	osteoarthritis
RMST	restricted mean survival time
